# Redox Homeostasis and Cellular Antioxidant Systems: Crucial Players in Cancer Growth and Therapy

**DOI:** 10.1155/2016/6235641

**Published:** 2016-06-21

**Authors:** Barbara Marengo, Mariapaola Nitti, Anna Lisa Furfaro, Renata Colla, Chiara De Ciucis, Umberto Maria Marinari, Maria Adelaide Pronzato, Nicola Traverso, Cinzia Domenicotti

**Affiliations:** ^1^Department of Experimental Medicine, General Pathology Section, University of Genova, 16132 Genova, Italy; ^2^Giannina Gaslini Institute, Genova, Italy

## Abstract

Reactive oxygen species (ROS) and their products are components of cell signaling pathways and play important roles in cellular physiology and pathophysiology. Under physiological conditions, cells control ROS levels by the use of scavenging systems such as superoxide dismutases, peroxiredoxins, and glutathione that balance ROS generation and elimination. Under oxidative stress conditions, excessive ROS can damage cellular proteins, lipids, and DNA, leading to cell damage that may contribute to carcinogenesis. Several studies have shown that cancer cells display an adaptive response to oxidative stress by increasing expression of antioxidant enzymes and molecules. As a double-edged sword, ROS influence signaling pathways determining beneficial or detrimental outcomes in cancer therapy. In this review, we address the role of redox homeostasis in cancer growth and therapy and examine the current literature regarding the redox regulatory systems that become upregulated in cancer and their role in promoting tumor progression and resistance to chemotherapy.

## 1. Pathophysiology of Reactive Oxygen Species and Antioxidant Defenses

**Figure 1:**
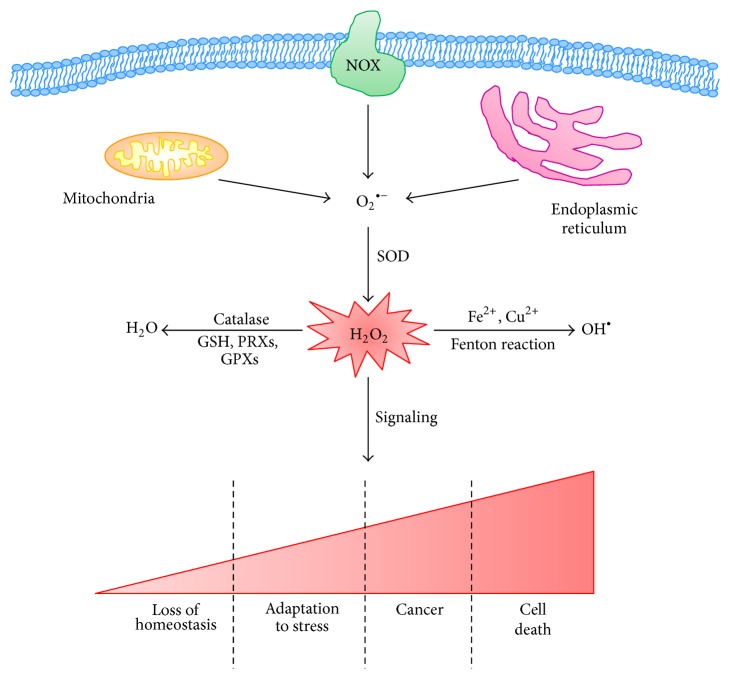
Redox homeostasis is a balance of ROS generation and elimination. Mitochondria, NAPH oxidase (NOX), and endoplasmic reticulum are the three major intracellular sources of ROS. Anion superoxide (O_2_^∙−^) is the principal form of ROS and can be rapidly converted into hydrogen peroxide (H_2_O_2_) by superoxide dismutase (SOD). H_2_O_2_ can be catalyzed to hydroxyl radical (OH^∙^) in the presence of Fe^2+^ or Cu^2+^ ions or be converted to H_2_O by catalase. The amount of H_2_O_2_ is decisive for the cell fate: low and intermediate levels of the peroxide stimulate loss of cell homeostasis and increased adaptation to stress leading to neoplastic transformation while high levels induce cell death.

Reactive oxygen species (ROS) are highly reactive molecules that are principally derived from the oxygen that is consumed in various metabolic reactions occurring mainly in the mitochondria, peroxisomes, and the endoplasmic reticulum. ROS include the superoxide anion (O_2_^∙−^) and hydroxyl radicals (OH^∙^) as well as nonradical molecules such as hydrogen peroxide (H_2_O_2_) [1]. H_2_O_2_ is the more stable and diffusible form of ROS, it is selectively reactive towards cysteine residues on proteins, and, in the low nanomolar range, it can control cellular signaling (Figure 1).

ROS are mainly produced by the mitochondrial respiratory chain and also by enzyme-catalyzed reactions involving NADPH oxidase (NOX), xanthine oxidase, nitric oxide synthase (NOS), arachidonic acid, and metabolizing enzymes such as the cytochrome P450 enzymes, lipoxygenase, and cyclooxygenase [2] (Figure 1).

The modulation of intracellular ROS levels is crucial for cellular homeostasis, and different ROS levels can induce different biological responses. At low and moderate levels ROS can act as signaling molecules that sustain cellular proliferation and differentiation and activate stress-responsive survival pathways [3]. For instance, ROS can stimulate the phosphorylation of protein kinase C (PKC), p38 mitogen-activated protein kinase (p38 MAPK), extracellular signal-regulated kinase (ERK)1/2, phosphoinositide 3-kinase/serine-threonine kinase (PI3K/Akt), protein kinase B (PKB), and JUN N-terminal kinase (JNK) [4–6]. ROS are also involved in the increased expression of antioxidant genes related to the activation of transcription factors such as the nuclear factor erythroid 2-related factor 2 (Nrf2), activator protein 1 (AP-1), nuclear factor *κ*B (NF-*κ*B), hypoxia-inducible transcription factor 1a (HIF-1a), and p53 [7–9].

At high levels, ROS promote severe cell damage and death. Cancer cells display elevated ROS compared to normal counterparts as the result of the accumulation of intrinsic and/or environmental factors. The more relevant factors include hypoxia, enhanced cellular metabolic activity, mitochondrial dysfunction, oncogene activity, increased activity of oxidases, lipoxygenases and cyclooxygenases, and the cross talk between cancer cells and immune cells recruited to the tumor site. Recent research has revealed that conditions inducing oxidative stress lead the neoplastic cells to develop powerful antioxidant mechanisms.

Several types of antioxidants play important roles in ROS homeostasis, including dietary natural antioxidants (e.g., vitamins A, C, and E), endogenous antioxidant enzymes (e.g., superoxide dismutase, catalase, glutathione peroxidase, glutathione reductase, and peroxiredoxins), and antioxidant molecules (e.g., glutathione, coenzyme Q, ferritin, and bilirubin).

*Superoxide Dismutases.* Superoxide dismutases (SOD) were the first characterized antioxidant enzymes [10] able to dismutate two O_2_^∙−^ anions into H_2_O_2_ and molecular oxygen. Three different types of SOD are expressed in human cells: copper-zinc SOD (CuZnSOD), which is present mainly in the cytoplasm, manganese SOD (MnSOD), located in the mitochondria, and extracellular SOD. It has been demonstrated that mice lacking MnSOD produce a massive oxidative stress and die perinatally [11] while CuZnSOD-deficient mice have persistent oxidative damage and develop hepatocellular carcinoma [12]. In addition, a variant allele of MnSOD has been associated with an elevated risk of prostate [13], lung [14], ovarian cancers [15], and non-Hodgkin's lymphoma [16].

*Catalase.* Catalase, a heme enzyme that catalyzes the reaction that converts two molecules of H_2_O_2_ to O_2_ and two molecules of H_2_O, is responsible for the detoxification of various phenols, alcohols, and hydrogen peroxide. Several epidemiologic studies have investigated the relationship between the mutations of catalase and human cancer but the results obtained are contradictory. In fact, a decreased catalase activity has been found both in blood samples and in tissues of breast cancer patients [17, 18] and in oral and pancreatic carcinomas [19, 20]. However, an increase in catalase levels has been reported in breast cancer tissue [21], malignant mesothelioma, and colorectal carcinoma [22, 23].

*Peroxiredoxins.* Peroxiredoxins (PRDXs) are a family of six isoenzymes able to reduce alkyl hydroperoxides and H_2_O_2_ to their corresponding alcohol or H_2_O. PRDXs are considered to be amongst the most important antioxidant enzymes, known to balance the production of cellular H_2_O_2_ which is essential for cell signaling and metabolism [24]. Under oxidative stress conditions, PRDXs are upregulated by Nrf2 activity and several studies have shown that the overexpression of PRDXs could either inhibit the development of cancer or promote growth of cancers [25].

In fact, PRDX1 interacts with the c-Myc oncogene and suppresses its transcriptional activity playing a tumor-suppressive role in breast cancer development [26, 27]. On the contrary, PRDX1 is associated with the promotion of oral, esophageal, lung, hepatocellular, and pancreatic carcinoma by upregulating heme oxygenase 1 and activating the NF-*κ*B pathway [28–31]. Moreover, also PRDX2 promotes colorectal carcinoma through upregulation of Wnt/*β* catenin and prostate cancer through upregulation of androgen receptor activity [32, 33]. Furthermore, several studies have demonstrated that the overexpression of PRDX1, PRDX2, and PRDX3 has an important role in many cases of drug resistance and that the therapeutic agents targeting these PRDXs are frequently studied for the treatment of cancer [34]. While PRDX3, PRDX4, and PRDX6 play a tumor-promoting role in the progression of many cancers [35–37], PRDX5, similar to PRDX1, has an antitumor effect in breast cancer development [38, 39].

*Thioredoxins.* Thioredoxins (Trxs) protect cells from oxidative stress by means of their 2-cysteine active site that reacts with ROS and is able to reduce oxidized proteins. They also serve as hydrogen donors to the thioredoxin-dependent peroxide reductases. Trx1, expressed in the cytoplasm and the nucleus, and Trx2, expressed in the mitochondria, are indispensable for cell survival [40]. Nuclear Trx1 has been shown to be overexpressed in* in situ *breasttumors [41], in melanoma, lung, colon, cervix, gastric, liver, and pancreatic carcinomas [42–45].

*Glutathione.* Glutathione (GSH) is the major cellular thiol protein, consisting of three amino acids glutamine, cysteine, and glycine, and it participates in antioxidant defense, in the detoxification of xenobiotics, and in many metabolic processes such as the synthesis of proteins and nucleic acids [46]. It is synthesized from L-glutamate, L-cysteine, and glycine in two consecutive steps, catalyzed by glutamate-cysteine ligase (GCL) and glutathione synthase (GS) [47]. GCL is considered the rate-limiting enzyme of GSH synthesis. While GSH loss, or a decrease in glutathione/glutathione disulphide ratio (GSH/GSSG), leads to an increased susceptibility to oxidative stress and to carcinogenesis, elevated GSH levels increase the antioxidant capacity of many cancer cells enhancing their resistance to oxidative stress [48]. Remarkably, the inhibition of GSH and Trx dependent pathways induces a synergistic cancer cell death, demonstrating the importance of these two antioxidants in favoring tumor progression [49]. Glutathione peroxidases (GPx) are another group of enzymes capable of reducing hydroperoxides, including lipid hydroperoxides, using GSH as a substrate and generating GSSG which is, once again, reduced by the specific enzyme glutathione reductase (GR). A proline-leucine substitution at codon 198 of human GPx has been associated with the increased risk of breast [50, 51], lung [52], and bladder cancer [53].

*Heme Oxygenase.* Heme oxygenase (HO)-1 is the first rate-limiting enzyme in the degradation of heme into biliverdin/bilirubin, carbon monoxide (CO), and free iron [54]. Normally expressed at low levels in most of the mammalian tissues, HO-1 expression is efficiently upregulated by the availability of its substrate heme and by different stress stimuli such as heavy metals, UV irradiation, ROS, nitric oxide, and inflammatory cytokines [55]. By increasing the availability of bilirubin, ferritin, and CO, with antioxidant and antiapoptotic properties, HO-1 is recognized as a key player in the maintenance of cellular homeostasis and in the adaptive response to cellular stressors [56]. For this reason, HO-1 activity is crucial in the protection of healthy cells, maintaining cell viability and counteracting ROS-mediated carcinogenesis as well [57]. However, the involvement of HO-1 in cancer cell biology has been proven [58] and the upregulation of HO-1 has been widely related to cancer cell metastatic and proangiogenetic potential and poor prognosis [59–61]. Nevertheless, the role of HO-1 seems to be strongly dependent on the types of tumor considered. For instance, in breast cancer cells, HO-1 activity reduces cell proliferation and favors the efficacy of certain drugs [62, 63]. Thus, it is important to note that the metabolic status of cancer cells may influence HO-1 expression that is dependent on different signaling pathways and transcription factors, suggesting a possible, but not completely understood, regulation of HO-1 [64]. In addition, it has been recently demonstrated that the response of myeloma cells to bortezomib could be due to the noncanonical functions of HO-1 which translocates to the nucleus where it plays a role in genetic instability, favoring cancer progression independently of its enzymatic activity [65]. Within this context, the nuclear localization of HO-1 has also been demonstrated to be involved in the gain of resistance to other chemotherapeutic agents such as imatinib in chronic myeloid leukemia [66]. As a whole, these findings open up a new scenario of the role of HO-1 in cancer cell biology.

## 2. Redox-Signaling Pathways Involved in Tumorigenesis and in Tumor Progression

In many tumors dysregulation of proliferation, apoptosis, and autophagy depends on the constitutive activation of redox-sensitive targets such as protein kinase C (PKC), protein kinase B (Akt), mitogen-activated protein kinases (MAPK), and ataxia telangiectasia mutated (ATM) kinase [135].

### 2.1. Protein Kinase C

Among redox-modulated signaling molecules playing a role in cancer, PKC may be activated by oxidative modifications of its enzymatic structure [136–138]. In this regard,* in vivo* and* in vitro* studies have demonstrated that high doses of prooxidant compounds cause PKC inactivation and proteolytic degradation while low doses induce the stimulation of the kinase activity [139–142].

For most PKC isoenzymes there is conflicting evidence as to whether they act as oncogenes or as tumor suppressors [143]. For example, the overexpression of PKC*α* has been demonstrated in prostate, endometrial, and high-grade urinary bladder carcinoma [144] while downregulation of PKC*α* has been described in basal cell carcinoma and colon cancers [145, 146]. Also PKC*β* overexpression is an early event in colon cancer development [147] and the transgenic overexpression of PKC*β*II induces hyperproliferation and invasiveness of intestine epithelial cells [148]. It has been reported that PKC*β* isoenzyme is responsible for the activation/phosphorylation of p66/shc, which can bind to cytochrome c and stimulate the generation of ROS [149]. Recent findings have demonstrated that PKC*α* plays a critical role in hepatocarcinoma development by inducing DUOX (a member family of NOX) expression and ROS production [150]. Moreover, also PKC*δ* has been shown to be implicated in NOX activation that* via* alterations of redox state influence retinoic acid-induced differentiation of neuroblastoma cells [151]. Likewise, PKC*δ* can act as either a positive or a negative regulator of tumor progression [152, 153]. Specifically, PKC*δ* may be overexpressed in colon cancers and downregulated in malignant gliomas, bladder carcinomas, and endometrial tumors [154]. Moreover, while the upregulation of PKC*δ*, in breast cancer patients, has been linked with the acquisition of resistance to tamoxifen [155] the overexpression of PKC*δ* in neuroblastoma cells induces apoptosis by sensitizing cells to etoposide [156].

### 2.2. PI3K/AKT

PI3K/AKT signaling contributes to tumorigenesis and to the expression of different cancer hallmarks. It facilitates the invasion and metastasis of cancer cells by promoting matrix metalloproteinase-9 (MMP-9) secretion [157] and by inducing the epithelial mesenchymal transition (EMT) [158] while it also increases telomerase activity and replication by activating telomerase reverse transcriptase (TERT) [159].

Furthermore, the PI3K/AKT signaling pathway has been found to activate NOX with production of ROS that on one hand may increase the genomic instability of cancer cells [160] and on the other hand may render cancer cells more sensitive to chemotherapy [161]. In addition, the upregulation of PTEN (phosphatase and tensin homolog deleted on chromosome 10), a tumor suppressor gene frequently deleted or mutated in many human cancers, has been demonstrated to reduce ROS generation by regulating the PI3K/AKT pathway [162]. ROS-dependent PTEN inactivation shifts the kinase-phosphatase balance in favor of tumorigenic tyrosine kinase receptor signaling through Akt, which inhibits apoptosis by phosphorylating and inactivating several targets, including Bad, forkhead transcription factors, and c-Raf and caspase-9 [163].

### 2.3. Apoptosis Signal-Regulating Kinase 1 (ASK1) and p38 MAPK

Apoptosis signal-regulating kinase 1 (ASK1) has been shown to act as a redox sensor by mediating the sustained activation of JNK and p38MAPK [164] resulting in apoptosis upon oxidative stress conditions [165]. In its inactive state, ASK1 is coupled to the reduced form of Trx 1 that induces its ubiquitination and degradation [166].

As above reported, p38 MAPK is able to inhibit tumor initiation by inducing apoptosis, by regulating cell cycle progression, and/or by inducing premature senescence of primary cells [167] This protein kinase contains four active cysteine residues that can be potentially oxidized. Although the activation of p38*α* is normally associated with antiproliferative functions [168, 169], several studies indicate that p38*α* can positively modulate cancer progression [170] as observed in malignant hematopoietic cells [171] and in other tumor cell lines [172]. Consistent with the prooncogenic role of p38MAPK, the inhibition of p38MAPK activity has been found to impair the proliferation and anchorage-independent growth of neuroblastoma cells [173].

### 2.4. Ataxia Telangiectasia Mutated (ATM) Kinase

A critical enzyme in maintaining genome stability is ATM, which can regulate DNA damage repair [174]. In fact, ATM upregulates the glucose-6-phosphate dehydrogenase to promote NADPH production and thus reduces ROS levels [175]. In cancer stem cells (CSCs), the ATM signaling pathway is highly active. In CD44+/CD24− stem-like cells, compared with other cell populations from breast cancer, the expression of ATM was significantly increased [176] and the employment of an ATM inhibitor reversed their resistance to radiotherapy, suggesting the importance of ATM signaling in CSC formation [176].

## 3. Role of Transcription Factors as ROS Modulators in Carcinogenesis and Cancer Progression

Many transcription factors are key players in regulating several pathways involved in carcinogenesis and cancer progression. Through their binding to the gene promoter regions, they can transactivate or repress the expression of antioxidant genes leading to the alteration in redox state and changes in proliferation, growth suppression, differentiation, and senescence.

### 3.1. p53

*p53* functions as a transcription factor able to activate or repress a large number of target genes that are involved in cell cycle control, DNA repair, apoptosis, and cellular stress responses [177]. It is kept at low levels by several E3 ubiquitin ligases, such as Mdm2, responsible for its degradation [178], and it is stabilized by posttranslational modifications such as phosphorylation, acetylation, and methylation [179, 180].

p53 has a controversial role in ROS regulation as it can promote both pro- and antioxidant responses [174].

Stress-induced p53 activation leads to the upregulation of several genes encoding ROS-generating enzymes, such as NQO1 (quinone oxidoreductase) [181] and proline oxidase (POX) [182], and redox-active proteins, including Bax and Puma. In particular, p53-induced ROS overproduction may be due to the overexpression of Puma, a critical mediator of mitochondrial membrane impairment [183], to the transcriptional activation of p67phox, a component of NADPH oxidase responsible for O_2_^∙−^ production [184] and to the action of p66Shc which oxidizes cytochrome c and affects mitochondrial permeability [149].

Moreover, the prooxidant activity of p53 has been found to be modulated by several genes named PIG1–13 (p53-inducible genes 1–13) which are able to encode redox-active proteins [181]. In particular, PIG1, a member of the galectin family, is involved in superoxide production; PIG3, homolog of NADPH-quinone oxidoreductase, is a potent ROS generator and PIG8, a human homolog of mouse E-24 gene, is a quinone able to regulate ROS [181].

In contrast, p53 is also able to transactivate different genes controlling antioxidant response in order to maintain ROS production at nontoxic levels [185]. In fact, p53 has been found to activate MnSOD expression* via* the direct recognition of the MnSOD human gene promoter [186] and to induce the expression of heme-oxygenase-1 (HO-1) by directly binding to the HO-1 promoter, favoring cell survival [187].

Another important antioxidant target of p53 is Tp53-induced glycolysis and apoptosis regulator (TIGAR) [188]. TIGAR encodes a protein that is similar to the glycolytic enzyme fructose-2,6-bisphosphatase, which degrades fructose-2,6-bisphosphate [189]. A decrease in fructose-2,6-bisphosphate levels inhibits the activity of the rate-limiting enzyme phosphofructokinase I (PFK1), thereby blocking glycolysis and promoting the shuttling of metabolites to the pentose phosphate pathway (PPP). By upregulating TIGAR, p53 amplifies PPP-mediated NADPH production that is required by glutathione reductase in order to convert GSSG to GSH. A third important antioxidant target of p53 is glutaminase 2 (GLS2) that converts glutamine to glutamate which is subsequently converted to GSH* via* GCLC and GCLM [190].

### 3.2. Nrf2

*Nrf2* is a transcription factor that controls not only the expression of antioxidants as well as phase I and phase II drug metabolizing systems, but also multidrug-resistance-associated protein transporters [58]. In a resting state, Nrf2 is sequestered in the cytoplasm through the binding with Keap1, responsible for Nrf2 ubiquitination and proteasomal degradation* via* Cul3. Oxidative/electrophilic stress causes a conformational change in Keap1-Cul3 by acting on specific residues in Keap1, leading to Nrf2 dissociation. Thus, Nrf2 translocates to the nucleus where it dimerizes with a small Maf protein and binds to the antioxidant response element (ARE) sequence within regulatory regions of a wide variety of target genes [191, 192]. In fact, Nrf2 is essential for the expression of stress-responsive or cytoprotective enzymes such as NQO1, SODs, HO-1, catalase, and Trx. In addition, Nrf2 activation regulates GSH levels and metabolism by inducing the expression of GCL, GS, GSH S-transferases (GSTs), GR, and GPx [193, 194].

Several mechanisms have been shown to be involved in the constitutive activation of Nrf2 in cancer cells, mainly gain-of-function mutations in Nrf2 and loss-of-function mutations in Keap1 [195–198]. Shibata et al. [199] have reported that Keap1 and Nrf2 mutations, in lung cancer, are responsible for the upregulation of ARE-modulated genes, which favor cancer promotion and/or progression [58]. Recently, these alterations of Keap1/Nrf2 pathway have been considered among the potential novel targets for the treatment of lung adenocarcinoma [200].

Among Nrf2 target genes glucose-6-phosphate dehydrogenase, phosphogluconate dehydrogenase, transketolase, and transaldolase I are responsible for NADPH and purine regeneration and then accelerate cancer cell proliferation [201]. Moreover, Nrf2 is directly involved in the basal expression of the p53 inhibitor Mdm2, through the binding to the ARE sequence located in the first intron of this gene, and inhibits cell death [202]. Cancer cells with high levels of Nrf2 have been shown to be less sensitive to etoposide, cisplatin, and doxorubicin [203] and our studies demonstrated that activation of Nrf2 and of its target genes plays a key role in the resistance of neuroblastoma cells to GSH depletion or proteasome inhibition [85, 204].

### 3.3. NF-*κ*B

The transcriptionfactor* NF-κB* plays a critical role in cell survival, proliferation, immunity, and inflammation [205]. In stimulated cells, I-*κ*B, an endogenous inhibitor able to retain NF-*κ*B in the cytoplasm, is phosphorylated by I-*κ*B kinase (IKK) which leads to I-*κ*B ubiquitination and proteasomal degradation and induces NF-*κ*B translocation to the nucleus where it can modulate the transcription of its target genes [206]. Morgan and Liu showed that ROS may regulate NF-*κ*B activation to express antioxidant genes coding MnSOD, Cu,Zn-SOD, catalase, Trx, GST-pi, HO-1, and GPx [207]. NF-*κ*B is also involved in the regulation of some enzymes catalyzing ROS production such as NOX2, xanthine oxidoreductase, NOS, and COX-2 [208].

NF-*κ*B activation leads to the development and/or progression of cancer by upregulating several genes involved in cell transformation, proliferation, and angiogenesis [209]. In this regard, it has been found that NF-*κ*B activation and ROS production promote the progression of hepatocellular carcinoma [210] and the initiation of colorectal cancer [211]. Moreover, as observed in high-risk myelodysplastic syndrome and in AML patients, NF-*κ*B activation, due to the constitutive activation of ATM [212], is critical for the survival of human leukemia cells [213] by increasing MnSOD activity, reducing ROS levels and inhibiting oxidative cell death.

### 3.4. HIF-1

Hypoxia-inducible factor (*HIF-1*) is a heterodimeric transcription factor composed of an *α*-subunit (HIF-1*α*) and a *β*-subunit (HIF-1*β*) [214]. The expression of HIF-1*α* is mainly regulated at the posttranslational level in an oxygen-dependent manner and is largely responsible for the regulation of HIF-1 activity [215].

It has been demonstrated that HIF-1*α* interacts with the HIF-1*β* and acts as a transcription factor able to induce the expression of genes involved in metabolic adaptation, such as hexokinase II (HK II) and pyruvate dehydrogenase kinase 1 (PDK1) [216], and the expression of genes involved in improving oxygen availability [217, 218] and shifting the glucose metabolism from mitochondrial oxidative phosphorylation to anaerobic glycolysis [219].

In addition, it has been demonstrated that ROS,* via* the modulation of PI3K/AKT and ERK pathways, are able to activate HIF-1 in hypoxic tumors [220]. In fact, HIF-1 overexpression correlates with poor outcomes in patients with head, neck, nasopharyngeal, colorectal, pancreatic, breast, cervical, bone, endometrial, ovarian, bladder, glial, and gastric cancers [9] and it is associated with refractiveness to conventional therapies [221].

## 4. ROS-Modulating Agents Undergoing Clinical Trials in Oncology

Several anticancer drugs are able to produce high levels of ROS leading to DNA damage and apoptosis [222, 223] that can be further stimulated by depleting cancer cell of GSH. The following compounds alter the intracellular redox state and induce cell death; for this reason some of them have been employed to improve the cytotoxic effects of conventional drugs (Table 1).

*L-Buthionine-S,R-sulfoximine* (BSO) induces oxidative stress by inhibiting GSH biosynthesis [67] and it synergizes with cytotoxic chemotherapeutic agents, including arsenic trioxide, cisplatin, doxorubicin, and melphalan [68]. Our studies have demonstrated that BSO-induced ROS overproduction and apoptosis of neuroblastoma cells is mediated by PKC*δ* activation [69–72] which is crucial for the sensitization of cancer cells to BSO and to etoposide [156]. In this context, BSO plus melphalan is currently undergoing clinical evaluation in children with neuroblastoma and in patients with persistent or recurrent stage III malignant melanoma [73].

*Menadione* (also known as vitamin K3) is a synthetic derivative of vitamins K1 and K2. The oxidative stress generated by menadione is dose-dependent and is due to GSH depletion capable of inducing cell death [74]. Moreover, a recent study reported that menadione analogues at submicromolar concentrations activate apoptosis of myeloid leukemia cells* via* the activation of ERK 1/2 and p38MAPK [75].* In vitro* investigations have led to the employment of menadione in different human trials in patients with gastrointestinal and lung cancer [76, 77].

*Imexon* is a prooxidant small molecule that depletes intracellular thiols generating oxidative stress and, subsequently, induces apoptosis [78]. Preclinical studies have demonstrated that imexon treatment increases nuclear Nrf2 levels and AP-1-DNA binding activity in myeloma cells and breast cancer cells [79]. These findings suggest that imexon leads to an adaptive response to oxidative stress involving upregulation of several antioxidant genes such as Nrf2 [79] and CuZnSOD [224]. The increased antioxidant gene expression and the enhancement of GSH levels in myeloma cell lines have been associated with the phenomenon of resistance to imexon [225].

Successful phase I trials have been completed in combination with cytotoxic chemotherapy in advanced breast, non-small cell lung cancer (NSCLC), prostate [80], and pancreatic [81] tumors. In addition, a phase II study has been carried out in patients with relapsed/refractory B-cell non-Hodgkin lymphoma [82].

*Disulfiram* is an acetaldehyde dehydrogenase inhibitor that induces apoptosis* via* GSH oxidation and proteasome inhibition [68, 83]. Preclinical studies have demonstrated that disulfiram-induced apoptosis of human melanoma cells [226] and of lymphoid malignant cells is mediated by JNK activation and Nrf2 and NF-*κ*B inhibition [84]. A phase I/II trial with disulfiram has recently been completed in patients with metastatic melanoma and other early-phase studies are ongoing in NSCLC and treatment-refractory liver tumors [68].

*Bortezomib* is a proteasome inhibitor that blocks inducible I-*κ*B degradation and consequently activates NF-*κ*B [86, 87]. It induces cell cycle arrest and apoptosis by preventing the degradation of p21/waf1, p53, and Bax [227]. Bortezomib has been extensively studied either alone or in combination with other agents for the treatment of multiple myeloma [86] and of chronic lymphocytic leukemia (CLL) [88]. In addition, bortezomib has been demonstrated to exert cytotoxicity by increasing ROS production [228] and, in this context, our recent studies have shown that bortezomib treatment of human neuroblastoma cells is less effective as a consequence of Nrf2-mediated HO-1 upregulation [85]. Moreover, it has been reported that bortezomib induces HO-1 activity in multiple myeloma* via* the endoplasmic reticulum stress pathway and that HO-1 nuclear translocation confers resistance to chemotherapy and induces genetic instability in cancer cells [65].

*NOV-002* is a product containing oxidized glutathione that alters the GSH/GSSG ratio and induces S-glutathionylation [91]. NOV-002-induced S-glutathionylation has been shown to have inhibitory effects on proliferation, survival and invasion of myeloid cell lines and significantly increases the efficacy of cyclophosphamide chemotherapy in a murine model of colon cancer [229]. NOV-002 has been most extensively studied with a phase III trial (NCT00347412) completed in the treatment of advanced NSCLC [92] and data is available from phase II trials in breast and ovarian cancers [230]. In a randomized phase II trial, NOV-002 in combination with standard chemotherapy has shown promising effects in patients with stage IIIb/IV of NSCLC [231]. Positive results were also obtained from a phase II trial in patients with neo adjuvant breast cancer therapy [93].

*Ezatiostat hydrochloride* (TLK199) is a GSH analogue that inhibits GST P1-1 leading to JNK/ERK activation and inducing apoptosis of malignant cells [94]. Treatment of leukemia cell lines with ezatiostat has been demonstrated to induce myeloblast differentiation without affecting myelopoiesis [94]. Ezatiostat has been evaluated in multiple phase I and phase II clinical trials in myelodysplastic syndrome (MDS) characterized by ineffective hematopoiesis presenting with anemia and, in some cases, neutropenia and thrombocytopenia [94].

*PX-12* (1-methylpropyl 2-imidazolyl disulfide) irreversibly inactivates Trx-1 which is overexpressed in many human cancers and it is associated with aggressive tumor growth and decreased patient survival [95]. Furthermore, the antitumor activity of PX-12 is also due to a reduction of VEGF in cancer patient plasma [95] and it can be synergistically enhanced after combination of PX-12 with 5-FU in HCC cells [96]. PX-12 has shown promising pharmacokinetics and pharmacodynamics in phase Ib trials in patients with advanced solid tumors refractory to chemotherapy [97].

*Dimesna* (BNP7787, disodium 2,2-dithio-bis-ethane sulfonate) is a novel chemoprotective disulfide compound that targets Trx and Grx which are overexpressed in many tumors [98, 99]. Dimesna has been employed in the treatment of various solid tumors, including ovarian carcinoma and NSCLC. In addition, it is currently undergoing phase III clinical trials (NCT00966914), in combination with first-line taxane and platinum chemotherapy, in patients with diagnosed or relapsed advanced (stage IIIB/IV) NSCLC adenocarcinoma.

*Motexafin gadolinium* (MGd) is a Trx inhibitor that reversibly accepts electrons from NADPH, NADH, GSH, and ascorbate, with subsequent electron transfer to molecular oxygen [232]. Preclinical studies have shown that MGd alone has a proapoptotic effect in multiple myeloma, non-Hodgkin lymphoma, and chronic lymphocytic leukemia [233]. MGd has been tested in a phase I trial in patients with locally advanced pancreatic or biliary cancers [97], and in a phase II trial in renal cell carcinoma [100] and in haematological malignancies [101].

*Arsenic trioxide* (As_2_O_3_) is an inorganic compound that has antiproliferative and apoptogenic effects on cancer cells by inducing oxidation of cysteine residues in GSH and thiol enzymes [68]. It has been approved by the European Medicines Agency and US Food and Drug Administration, for induction and consolidation of chemotherapy in adults with relapsed/refractory acute promyelocytic leukemia (APL). Moreover, As_2_O_3_, in combination with disulfiram, is being evaluated as a second-line therapy in phase I trials (NCT00571116) in patients with metastatic melanoma.

## 5. Conclusions

**Figure 2:**
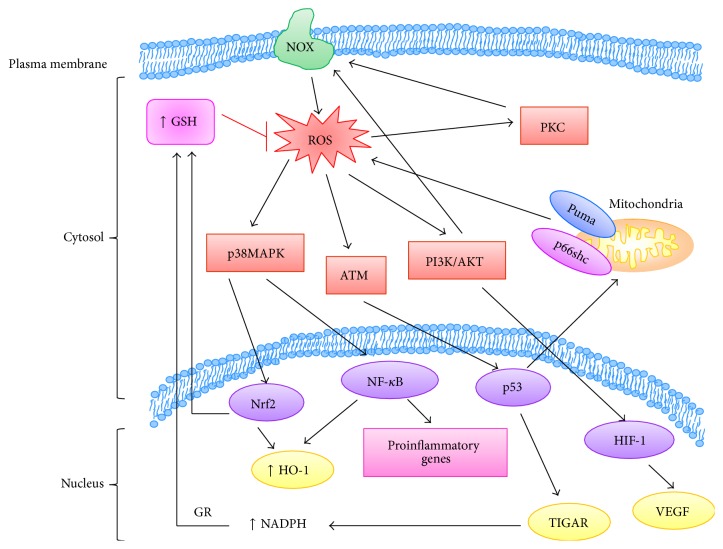
Redox-signaling pathways that are involved in cancer growth and progression. Cancer cells escape cell death and damage induced by high ROS levels by increasing their antioxidant defenses such as GSH that contribute to lower the amount of ROS. ROS are produced by NOX in the plasma membrane and by mitochondria, and at low levels they act as second messengers by activating many protein kinases (PI3/Akt, p38 MAPK, and ATM) and transcription factors (Nrf2, NF-*κ*B, p53, and HIF-1) able to contribute to cancer cell survival by stimulating cell proliferation, inflammation, and angiogenesis. GR, glutathione reductase.

The modulation of oxidative stress is considered an important factor in the development of cancer and in the response of tumor cells to therapy [189]. As shown in this review, high ROS levels in cancer cells are a consequence of alterations in cellular metabolism and their overproduction is counteracted by elevated defense mechanisms (Figure 2).

Among antioxidants, GSH is essential for maintaining a correct redox balance, has a crucial role in the protection of cancer cells from oxidative stress, and ensures cell survival in both hypoxia and nutrient deprivation that are present in solid malignant tumors [48]. For this reason, combinations of GSH antagonists or other antioxidant inhibitors with radio or chemotherapy may be useful for killing cancer cells. This “epigenetic-genetic” therapeutic approach is in sharp contrast to the conventional strategy of targeting oncogenes and oncosuppressors, an approach that has turned out to be uneffective also for the frequent gene mutations.

As reported in this review, many of these genes are redox-sensitive transcription factors that are involved in proliferation, angiogenesis, and metastasis and are able to induce a common set of cell stress adaptive responses, thus providing a survival advantage.

Therefore, the redox-signaling pathways underlying these adaptations may represent the most critical weak point in many cancers and the signaling molecules that mediate these changes could be the next important targets for future anticancer drug discovery research.

Recently, as summarized in Table 2, many clinical trials with modulators of kinases or transcription factors associated with conventional therapy are ongoing. Although the results of some of these combined strategies seem to be promising, further studies are needed in order to identify specific markers for a more personalized therapy and to minimize the side toxic effects.

## Figures and Tables

**Table 1:** ROS modulating drugs undergoing clinical trials in oncology.

Drug	Mechanism of action	Cancer type	Outcome	Ref.
L-Buthionine-sulfoximine	Inhibits GSH synthesis; activates PKC*δ*	Neuroblastoma Melanoma	Efficacious *in vitro*	[67–73]
Menadione	Depletes GSH; activates ERK1/2 and p38MAPK	Gastrointestinal and lung cancer	Under clinical trial	[74–77]
Imexon	Depletes intracellular thiols; increases AP-1 and Nrf2-DNA binding activity	Advanced breast cancer; NSCLC; prostate and pancreatic tumors	Efficacious	[78–82]
Disulfiram	Oxidizes GSH and inhibits proteasome; activates JNK; inhibits Nrf2 and NF-*κ*B	Metastatic melanoma; liver cancer	Under clinical trial	[68, 83, 84]
Bortezomib	Inhibits proteasome activity; activates NF-*κ*B; activates Nrf2 and upregulates HO-1	Myeloma, leukemia, AML, myelodysplastic syndrome, neuroblastoma, prostate cancer	Under clinical trial	[85–90]
NOV-002	Oxidizes GSH and induces S-glutathionylation	NSCLC; breast and ovarian cancer	Efficacious	[91–93]
Ezatiostat	Inhibits GST-P1 and activates JNK/ERK	Myelodysplastic syndrome	Under clinical trial	[94]
PX-12	Inactivates Trx-1	Advanced solid tumors	Efficacious	[95–97]
Dimesna	Targets Trx and Grx	Ovarian carcinoma, NSCLC	Efficacious	[95, 98, 99]
Motexafin gadolinium	Inhibits Trx	Pancreatic, biliary and haematological cancer, renal carcinoma	Under clinical trial	[97, 100–102]
Arsenic trioxide	Oxidizes GSH and thiol enzymes	APL, melanoma	Efficacious	[68]

**Table 2:** Modulators of redox signaling pathways employed in combination with anticancer agents and their effects.

Drug	Mechanism of action	Cancer type	Outcome	Ref.
Trametinib	MEK inhibitor	Melanoma	Efficacious	[103]
Selumetinib	MEK inhibitor	Thyroid, ovarian cancer	Efficacious	[104–106]
Tamoxifen	PKC inhibitor	Gliomas, breast cancer	Efficacious	[107–111]
Perifosine	Akt, MAPK and JNK inhibitor	Haematologic tumors, myeloma	Efficacious	[112–116]
Sulfasalazine	NF-*κ*B inhibitor	Colorectal cancer	Efficacious	[117, 118]
Nelvinavir	Decreases HIF-1*α*	Adenoid cystic carcinoma, pancreatic cancer, NSCLC	Efficacious	[119–122]
Topotecan	HIF-1 and Topoisomerase I inhibitor	Endometrial and cervical cancer	Efficacious	[123, 124]
Aprinocarsen	Antisense oligonucleotide against PKC-*α*	Lymphoma, breast cancer	Contrasting results	[125–127]
Midostaurin	Multitarget inhibitor of PKCs, VEGFR2, PDGFR	AML, melanoma	Contrasting results	[128, 129]
MK-2206	Akt and PI3K inhibitor	Gastric, pancreatic and breast cancer	Under clinical trial	[130]
Serdemetan	mdm2 inhibitor	Refractory solid tumors	Under clinical trial	[131]
PRIMA-1 and PRIMA-1^MET^	Reverse the oncogenic properties of mutant p53	Ovarian cancer	Under clinical trial	[132, 133]
AMG 232	mdm2-p53 interactions inhibitor	Melanoma, myeloma, myeloid leukemia	Under clinical trial	[134]
